# PKIS: computational identification of protein kinases for experimentally discovered protein phosphorylation sites

**DOI:** 10.1186/1471-2105-14-247

**Published:** 2013-08-13

**Authors:** Liang Zou, Mang Wang, Yi Shen, Jie Liao, Ao Li, Minghui Wang

**Affiliations:** 1 Department of Electronic Science and Technology, University of Science and Technology of China, Hefei 230027, China; 2 Research Centres for Biomedical Engineering, University of Science and Technology of China, Hefei 230027, China

## Abstract

**Background:**

Dynamic protein phosphorylation is an essential regulatory mechanism in various organisms. In this capacity, it is involved in a multitude of signal transduction pathways. Kinase-specific phosphorylation data lay the foundation for reconstruction of signal transduction networks. For this reason, precise annotation of phosphorylated proteins is the first step toward simulating cell signaling pathways. However, the vast majority of kinase-specific phosphorylation data remain undiscovered and existing experimental methods and computational phosphorylation site (P-site) prediction tools have various limitations with respect to addressing this problem.

**Results:**

To address this issue, a novel protein kinase identification web server, PKIS, is here presented for the identification of the protein kinases responsible for experimentally verified P-sites at high specificity, which incorporates the composition of monomer spectrum (CMS) encoding strategy and support vector machines (SVMs). Compared to widely used P-site prediction tools including KinasePhos 2.0, Musite, and GPS2.1, PKIS largely outperformed these tools in identifying protein kinases associated with known P-sites. In addition, PKIS was used on all the P-sites in Phospho.ELM that currently lack kinase information. It successfully identified 14 potential SYK substrates with 36 known P-sites. Further literature search showed that 5 of them were indeed phosphorylated by SYK. Finally, an enrichment analysis was performed and 6 significant SYK-related signal pathways were identified.

**Conclusions:**

In general, PKIS can identify protein kinases for experimental phosphorylation sites efficiently. It is a valuable bioinformatics tool suitable for the study of protein phosphorylation. The PKIS web server is freely available at http://bioinformatics.ustc.edu.cn/pkis.

## Background

Reversible protein phosphorylation, which is one of the most common post-translation modifications in eukaryotes, is involved in various cellular processes including regulation of metabolism [1], DNA repair [2], and cellular differentiation [3]. It plays an especially dominant role in signal transduction in biological systems [4,5]. Kinase-specific phosphorylation data including substrate sites (P-sites) and the corresponding protein kinase is the root of reconstruction of signal transduction networks and is widely used in different fields of biomedicine, especially in the identification of potential drug targets [6,7]. For this reason, precise annotation of phosphorylated proteins is key to further research regarding phosphoproteomes.

In recent years, considerable efforts have been devoted to experimental and computational identification of phosphorylation data. Historically, phosphorylation sites were discovered mainly using low-throughput technology [8]. However, these biotechniques, such as ^32^P-labeling and degenerate peptide library screening, are costly, labor-intensive, and time consuming [9,10]. With recent developments in mass spectrometry, experimentally verified phosphorylation data have accumulated rapidly. For example, Wiśniewski et al. identified nearly 12,035 unique P-sites in 4,579 mouse brain proteins using mass spectrometry [11]. However, this high-throughput technology cannot provide information regarding the protein kinases that catalyze phosphorylation substrates. Systematically matching these P-sites to specific kinases experimentally is not currently feasible [12]. This limits the amount of protein kinase information that can be made available in phosphorylation databases. For example, a well-known database of experimentally verified phosphorylation data in eukaryotes, Phospho.ELM, currently lists 3,151 phosphorylation sites with corresponding kinase information [13]. However, this accounts for less than 12% of the total of 27,404 human phosphorylation sites deposited in this database. The increasingly large gap between experimentally verified phosphorylation data and protein kinase information hampers studies on protein phosphorylation and signal transduction pathways. Existing kinase-specific phosphorylation site prediction tools, such as PPSP [14], KinasePhos 2.0 [15], Musite [16] and GPS2.1 [17], may generate kinase information for experimentally verified phosphorylation sites, but they focus on predicting novel phosphorylation sites and therefore show less than optimal performance for other purposes.

To address these limitations, this study presents a novel bioinformatics tool called the protein kinase identification server (PKIS). It is designed for the identification of protein kinases that act at known P-sites with high specificity. Human phosphorylation data was retrieved from the Phospho.ELM database and used to train the kinase identification models by incorporating the composition of monomer spectrum (CMS) with SVMs [18]. Comprehensive analysis shows that CMS encoding performs better than binary encoding in identifying protein kinases for known P-sites. The results of performance evaluation show that PKIS is more powerful than widely used P-site prediction tools.

## Results

### Prediction performance in different window sizes

Previous studies have demonstrated that the side chains of amino acids surrounding P-sites influence the phosphorylation process, including contacts with kinases. In this way, the sequence surrounding a P-site plays a vital role in determining which kinase catalyzes the corresponding phosphorylation substrate. However, the residues enclosing the P-sites in the linear sequence may not be adjacent spatially, and distinguishing the residues surrounding the P-sites exactly for all the phosphorylated proteins experimentally is difficult and time-consuming [8]. For these reasons, all residues within 30 aa (amino acid) of the P-sites were selected for further examination.

**Figure 1:**
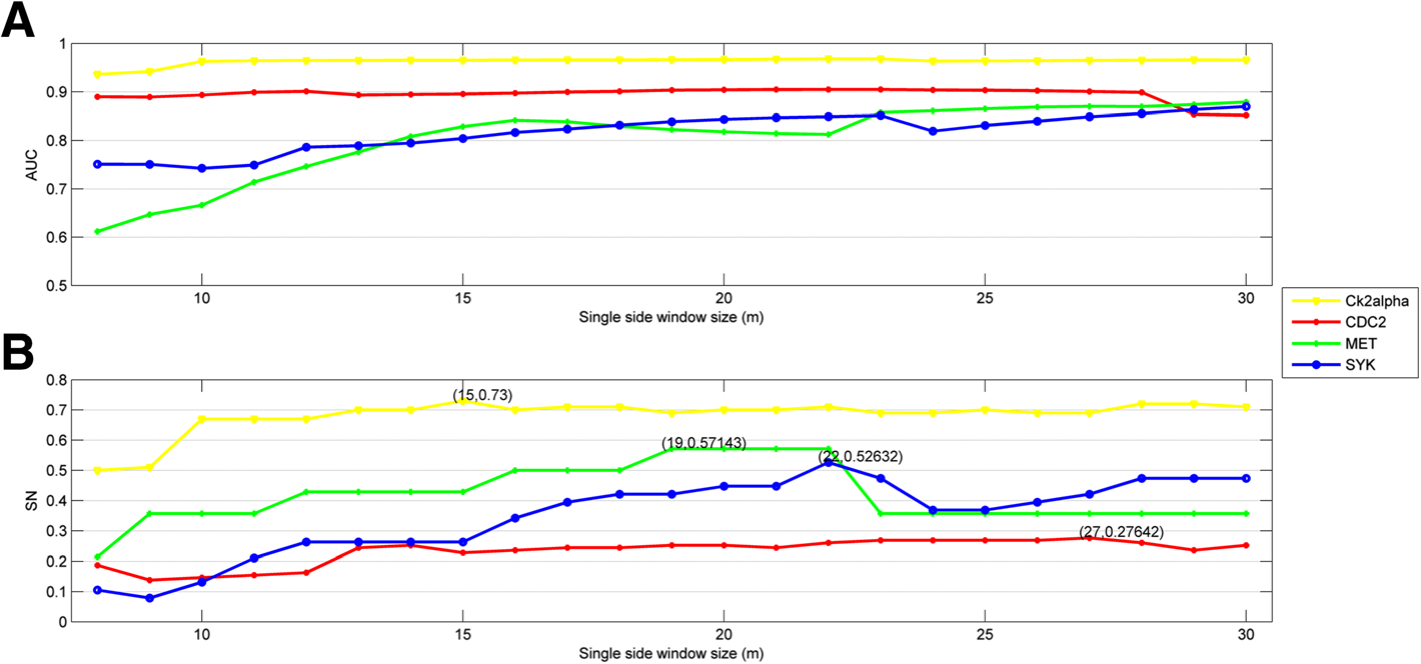
**Prediction performance of models with different single-side window sizes *m*. (A)** The escalating trend for AUC with the improvement of *m*. The slope of the left side is larger than that of the right. **(B)** The optimal *m* for kinases is diverse. Sensitivity was evaluated when the corresponding specificity was greater than or equal to 99%.

In light of kinases’ specificity in protein phosphorylation, it does not make sense to use a fixed window size for all kinases [19]. For this reason, LOOCV was applied to evaluate performance with respect to the different window sizes used in CMS encoding. Two Ser/Thr kinases, CK2alpha and CDC2, and two Tyr kinases, MET and SYK, served as examples. As shown in Figure 1A, increasing the window size is generally associated with better AUC, especially when the window size is small. For example, the AUC for MET kinase is 0.611 when *m* is equal to 8, but it soars to 0.842 when *m* increases to 16. The improvement in AUC begins to slow down as window size increases beyond 16, finally stopping at the maximum value of *m*. This indicates that all the residues implicated in the phosphorylation process have been taken into account. It is of note that there are some fluctuations in improvement as the window size increases, probably due to complex interactions between residues.

To identify protein kinases confidently, the specificities of the SVM models in PKIS were all required to be at least 99.0%, which meant that the expected rate of false positive results was not larger than 1.0%. Then changes in sensitivity at different window sizes were examined (Figure 1B). For MET kinase, the best sensitivity (0.57) is obtained when *m* is equal to 19. For this reason, this optimized window size was used to build the SVM model for MET kinase in PKIS.

### Evaluation of CMS encoding

**Figure 2:**
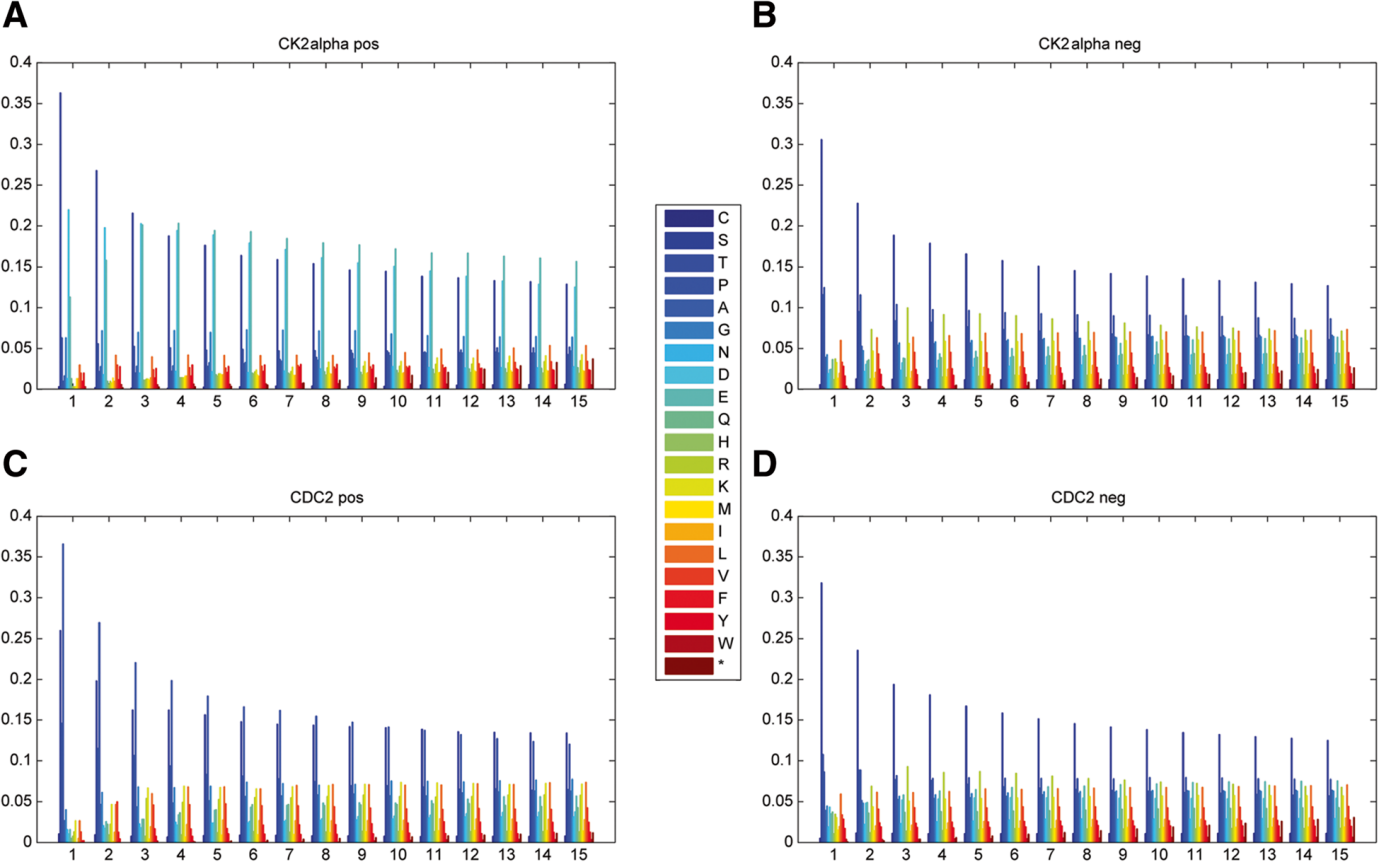
**Difference of amino acid distributions in positive and negative data.** Panels **(A)** and **(B)** represent distinct amino acid distribution patterns in CK2 alpha’s positive and negative datasets, respectively. Panels **(C)** and **(D)** represent different amino acid distribution patterns in CDC2’s positive and negative datasets, respectively. The X-axis represents the single side window size *m.*

An essential part of developing a protein kinase identification system is the encoding of the side chains surrounding the P-sites. A good, high-performance encoding strategy may also provide insight into the biological mechanism of phosphorylation. First, we examined the features encoded by CMS that represent different amino acid compositions under a series of increased window sizes. Amino acid compositions were found to be largely different for the positive and negative data in most of the kinases. For example, Figure 2 illustrates the distributions of amino acids for CK2alpha and CDC2 kinase in different window sizes. Asp and Glu are enriched in the side chains of P-sites catalyzed by CK2alpha, whereas Arg is enriched in the side chains of P-sites catalyzed by other kinases (Figure 2A and Figure 2B). Likewise, Pro is only enriched in the substrates of CDC2 kinase that is considered as a proline-directed kinase [20] (Figure 2C and Figure 2D). Multivariate analysis of variance (MANOVA) of the CMS encoded features was performed for evaluation of statistical differences in amino acid composition. As shown in Additional file 1: Table S1, a total of 50 kinases pass the statistical test, as MANOVA requires that the sample size be larger than the number of variates. The majority of these kinases (30 Ser/Thr kinases, 6 Tyr kinases) exhibit significant differences (*P*-value <0.05) in CMS encoded features, which is consistent with the disparity of amino acid compositions between positive and negative data. These results demonstrate that CMS-encoded features are useful for the determination of which kinase catalyzes the corresponding phosphorylation process.

**Figure 3:**
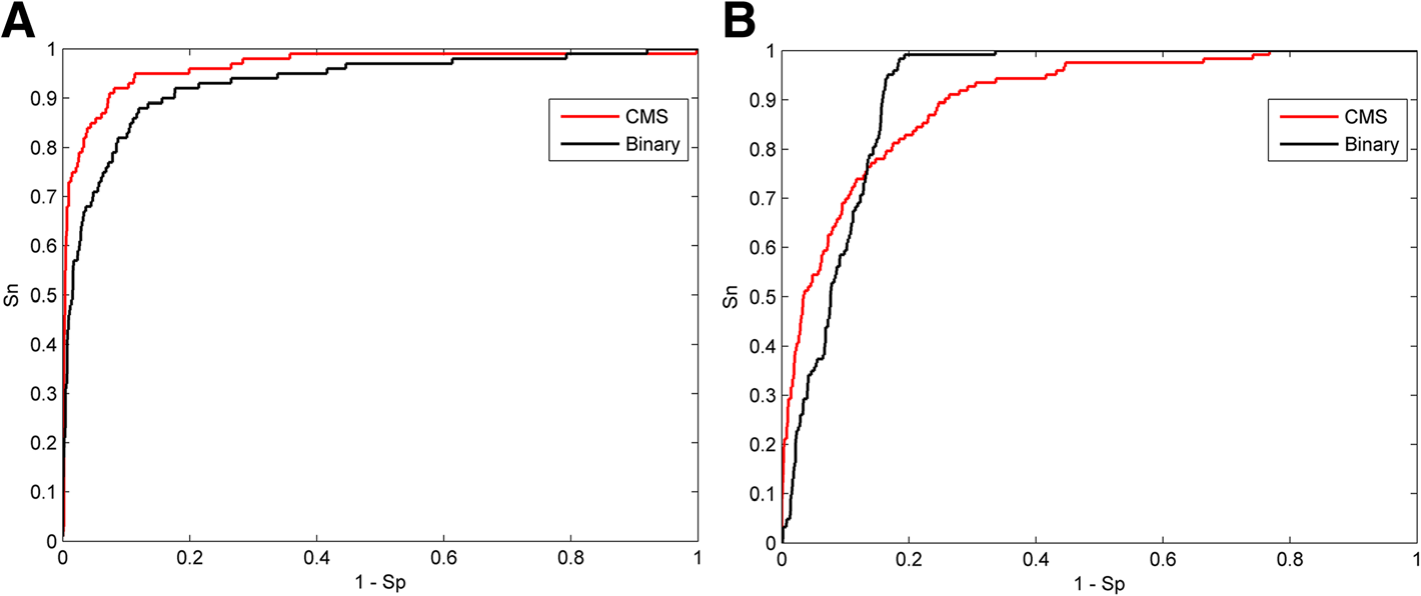
**Performance of two sequence encoding strategies: CMS and binary encoding. (A)** Performance of CK2 alpha models using the CMS and binary encoding strategies. **(B)** Performance of CDC2 models using CMS and binary encoding strategies. The red lines represent the CMS method and the black lines represent the binary method.

Another encoding strategy, binary encoding was also investigated. Binary encoding is widely used in bioinformatics studies of protein phosphorylation. In binary encoding, a 21-dimensional binary vector represents each amino acid and an end-of-sequence marker. Phosphorylation data were encoded based on CMS and binary strategies and the performance of these two methods was evaluated using LOOCV. The ROC curves for CK2alpha and CDC2 kinase were used as examples (Figure 3). Accompanied with larger AUC for CK2alpha kinase, CMS encoding shows consistently better performance than binary encoding (Figure 3A). For CDC2 kinase, crossed ROC curves with similar AUCs are observed for both encoding methods. However, CMS encoding demonstrates a significant increase in sensitivity (27.6%) with a high level of specificity (99.1%), when compared to binary encoding (4.9%). In addition, these two encoding strategies were compared for other kinases and results showed CMS encoding had a noticeable advantage over binary encoding for a majority of kinases. Taken together, it is concluded that CMS is a superior encoding strategy in identifying protein kinases.

### Comparing with kinase-specific P-site prediction tools on the Phospho.ELM database

The performance of PKIS was evaluated and compared to three widely used kinase-specific P-site prediction tools: KinasePhos2.0 [15], Musite [16], and GPS2.1 [17]. It should be pointed out that none of these tools provide an option for unbiased evaluation of performance (e.g. LOOCV). In this case, we had to use all human phosphorylated proteins in Phospho.ELM database as testing data to assess their performance. These results were biased, because the P-sites in the Phospho.ELM database were also used for model training by these tools [15-17]. This inevitably lead to over-estimations of performance. Additionally, the performance of PKIS was examined using LOOCV, which can accurately reflect the true performance of the proposed method.

**Figure 4:**
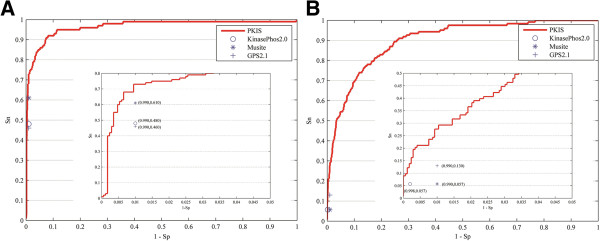
**Comparing with kinase-specific P-site prediction tools: KinasePhos2.0, Musite, and GPS2.1 at high specificities.** Panel **(A)** depicts the performance of the tool in CK2 alpha kinase and **(B)** illustrates the performance in CDC2 kinase. The ROC curves of PKIS are plotted in red solid lines.

To evaluate performance at high specificity, a threshold for decision scores and probabilities returned by P-site prediction tools was used to ensure that specificity levels fell as closely to 99.0% as possible. As shown in Figure 4A, for CK2alpha kinase, the sensitivities of KinasePhos2.0, Musite, and GPS2.1 are found to be 48.0%, 61.0%, and 46.0%, respectively, but PKIS shows better sensitivity, giving a value of 73.0% at the same level of specificity. Likewise, for CDC2 kinase, PKIS shows the best performance at specificity greater than or equal to 99% (Figure 4B). These results suggest that PKIS is superior to these P-site prediction tools at a high specificity. This is corroborated by the ROC curves of different methods (Additional file 2: Figure S1). In addition, the cross-classifying specificities for Ser/Thr kinases (Additional file 3: Table S2) and Tyr kinases (Additional file 4: Table S3) shows that the kinase models in PKIS can generally achieve very high cross-classifying specificities, suggesting that they can correctly recognize P-sites catalyzed by other protein kinases. In a few cases, the cross-classifying specificities are relatively low. For example, the specificity of ZAP70 kinase in SYK model is 82% because these two kinases are in the same kinase family and share similar substrate specificity [21].

### Performance of the PKIS web server on testing data

To assess the performance of the PKIS web server, the phosphorylation data from high-resolution maps of the human phosphorylation network were extracted and a testing dataset of experimentally identified kinase-substrate pairs was constructed [22]. To make results independent of any training data used by the PKIS web server, phosphorylation sites recorded in Phospho.ELM database were carefully checked and removed from the testing data. The results in Table 1 show that consistent with the LOOCV results, the PKIS web server shows high specificity in identifying protein kinases on the testing data. For example, the LOOCV specificity of PKIS for CK2 alpha kinase is 99.0% and the specificity for the testing data is found to be 99.1%. For comparison, the performance of protein kinase identification was also evaluated utilizing GPS2.1, KinasePhos2.0, Musite, and PPSP at similar specificities. PKIS demonstrates sensitivity superior to that of other P-site prediction tool. For the PKCa kinase, the sensitivity of PKIS is 37.3%, but the sensitivity of GPS2.1, KinasePhos2.0, Musite, and PPSP is 0%, 1.7%, 0%, and 8.5%, respectively. In addition, the cross-classifying performance of two Ser/Thr kinases was examined. These kinases, Erk2 and p38a (both in MAPK subfamily), have similar substrate motifs and significant proline enrichment at the +1 and −2 positions (Additional file 5: Figure S2). PKIS demonstrates performance consistently superior to that of GPS 2.1 and PPSP (Additional file 6: Table S4), with cross-classifying specificities of 93.9% and 94.2%, respectively. Among Tyr kinases, LCK and FYN (both in SRC subfamily), which exhibit similar substrate motifs with no prominent amino acid preference at any of the positions flanking the P-sites (Additional file 5: Figure S2), PKIS also outperforms all the other methods evaluated in this study (Additional file 6: Table S4). Taken together, these results demonstrate that as compared to P-site prediction tools, PKIS exhibits superior performance in the high-specificity identification of protein kinases, even if the protein kinases examined contain similar substrate motifs. In this way, PKIS are found to be especially suitable for large-scale phospho-proteomics studies and systematic investigations of signaling pathways.

**Table 1:** Comparison of PKIS with kinase-specific P-site prediction tools on testing data

**Kinase**	**PKIS**		**GPS2.1**		**Musite**		**KinasePhos2.0**		**PPSP**	
	**Sn**	**Sp**	**Sn**	**Sp**	**Sn**	**Sp**	**Sn**	**Sp**	**Sn**	**Sp**
Erk2 (MAPK1)	13.9%	97.6%	5.7%	97.2%	4.4%	97.4%	3.8%	97.4%	13.9%	97.6%
p38a (MAPK14)	13.5%	97.3%	0.0%	96.3%	8.1%	96.6%	0.0%	97.3%	5.4%	97.3%
CK2alpha	60.7%	99.1%	58.3%	99.0%	49.1%	99.1%	35.6%	99.1%	53.4%	99.0%
CDC2	37.5%	93.3%	12.5%	92.0%	0.0%	90.3%	0.0%	93.2%	12.5%	93.2%
PKCa	37.3%	99.8%	0.0%	99.4%	0.0%	99.6%	1.7%	99.4%	10.2%	99.7%
SYK	45.0%	93.0%	25.0%	93.0%	NA	NA	35.0%	94.4%	45.0%	93.0%
LCK	40.0%	97.4%	26.7%	92.1%	6.7%	93.4%	20.0%	96.1%	40.0%	97.4%
FYN	23.5%	94.6%	11.8%	94.6%	5.9%	90.5%	23.5%	94.6%	23.5%	94.6%

NA: This tool could not predict whether a residue had been phosphorylated by the corresponding kinase or not.

### A case study

Increasing knowledge of P-sites and their corresponding protein kinases is critical to reconstructing signal transduction pathways. In the present study, PKIS was used to identify P-sites phosphorylated by SYK kinase, which has been reported to mediate various cellular processes [23]. There are a total of 38 P-sites across 17 proteins that are phosphorylated by SYK kinase, as indicated by Phospho.ELM. By applying PKIS to all verified P-sites without kinase information, 14 new substrates of SYK kinase and 36 potential P-sites were discovered. These two datasets were then combined and enrichment analysis was performed employing DAVID to identify relevant pathways [24,25]. As shown in Table 2, 6 KEGG pathway categories are found to be significantly enriched and to have Benjamini *P*-values below 0.05. The most significant pathway is associated with natural-killer-cell-mediated cytotoxicity (Benjamini *P*-value 1.27E-6). Cytotoxicity mediated by natural killer cells is a very important immune response, playing both anti-viral and anti-tumor roles [26]. In this pathway, 9 proteins are found to be significantly enriched, and 2 of them (UniProt ID: P15498, P78314) are not included in the Phospho.ELM database. Careful mining of the literature show that the relevant proteins are known to be phosphorylated by SYK [27-29]. In this way, PKIS facilitates the discovery of novel relationships between protein kinases and their substrates in signaling pathways. Two other SYK-related pathways, B cell receptor signaling pathway and the pathogenic Escherichia coli infection pathway, are discovered using combined datasets. These pathways would have been missed by DAVID without the new substrates identified by PKIS. Apart from P15498, there are 3 more proteins (UniProt ID: P15391, Q13509, P68366) within these two pathways that are not included in Phospho.ELM but are identified as substrates of SYK. Previous studies have confirmed that all of them are phosphorylated by SYK [30-32]. These results clearly demonstrate the utility of PKIS in identifying protein kinases for experimentally verified P-sites, which can facilitate the identification of new substrates for protein kinases and the discovery of novel signal transduction mechanisms.

**Table 2:** Significant KEGG pathways enriched in the combined dataset

**Term**	**Count(**^**2**^**)**	***P*-value**	**Benjamini *P*-value**
Natural-killer-cell-mediated cytotoxicity	9 (2)	2.71E-08	1.27E-06
Fc-gamma-R-mediated phagocytosis	6 (1)	3.29E-05	7.72E-04
Fc epsilon RI signaling pathway	5 (1)	2.52E-04	2.95E-03
B cell receptor signaling pathway ^1^	5 (2)	2.16E-04	3.38E-03
Pathogenic *Escherichia coli* infection ^1^	4 (2)	1.54E-03	1.44E-02
ErbB signaling pathway	4 (0)	5.15E-03	3.97E-02

^1^ Term not found when only kinase-specific phosphorylated data was used in Phospho.ELM.

^2^ The number of Syk’s substrates predicted by PKIS at high specificity.

### Web interface

PKIS is freely accessible to users at the following web address: http://bioinformatics.ustc.edu.cn/pkis/. Users can submit phosphorylated proteins with verified P-sites and select all or some of the 56 predictive models available for protein kinase identification. In Additional file 7: Figure S3, the predicted results are presented as a table in which each row represents a unique kinase-substrate pair. To better understand the substrate binding preferences of each protein kinase, the CMS logo representing the substrate’s specificity is also provided in the predicted results. Datasets for all 56 kinases, including corresponding accession numbers and protein sequences can be downloaded from http://bioinformatics.ustc.edu.cn/pkis/download.html.

## Conclusions and discussions

Protein kinase identification is attracting significant attention due to the large number of P-sites discovered using high-throughput technologies. In the present study, a novel kinase identification web server was developed based on CMS encoding strategy and SVMs. In addition, to achieve optimal performance we generated specific negative data for SVM training in that different negative dataset construction strategies can bring about significantly different performance with respect to the classification problems [33]. The results showed PKIS outperformed many existing P-site prediction tools for the identification of protein kinases. However, there is still room for further improvement. The system showed limited identification performance for a few kinases. Protein phosphorylation is a highly complex biological process occurring *in vivo*. As such, the primary sequences around the potential P-sites may be not sufficient to indicate the corresponding protein kinase. The performance of this system may be enhanced by incorporating more biological information, such as protein functional domains and subcellular localization. Currently, kinase-specific phosphorylation data for other organisms are still sparse. However, with rapidly accumulated phosphorylation data, it may be possible to develop a platform that can be used to accurately identify protein kinases in multiple organisms.

## Methods

### Data collection

All 37,145 phosphorylation instances in humans were extracted from the latest version of Phospho.ELM (9.0). After excluding redundant records, 27,404 P-sites were recognized in 5,374 proteins, including 3,151 kinase-substrate pairs. These phosphorylation sites and their kinase information were used for further analysis. For each kinase, the corresponding phosphorylation instances were used as positive data (+). Negative data (−) were comprised of phosphorylation events catalyzed by other kinases, instead of non-phosphorylation sites that were used by P-site prediction tools. To ensure reliable results, a total of 56 kinases with more than 10 positive instances were selected. See Additional file 8: Table S5 summarizes the statistics of all these kinases.

### Feature extraction

In this study, sequence information was encoded using an efficient encoding strategy called CMS [18]. As a part of the CMS, monomer spectrum (MS) represents the amino acid composition and the corresponding feature value is the occurrence frequency of each amino acid in a certain window. For example, for the peptide CADKSPEQSPDAEYPTH, the resulting MS feature vector is 1, 2, 1, 3, 2, 0, 0, 2, 2, 1, 1, 0, 1, 0, 0, 0, 0, 0, 1, 0, 0. For a protein sequence with a single side window size of *m*, CMS incorporates different MS vectors under a series of window size from 3 to 2*m+1. Unlike the MS encoding strategy, this reflects the occurrence of the amino acids in certain positions and therefore provides more sequential information than amino acid composition for given window size. Additional file 9: Figure S4 shows the differences in CMS and MS encoding strategies.

### Classification and evaluation

The kinase identification system was constructed by incorporating SVMs with CMS features. LIBSVM, a public SVM library, was selected for training classification models [34]. The radial basis function (RBF) was used as the kernel function. The cost (*c*) value and the gamma (*γ*) value were optimized and used to enhance the strength of the classifiers. Leave-one-out cross validation (LOOCV, also called the Jack-knife cross validation), which is the most objective and rigorous method of assessing a classifier, was used to evaluate the performance of PKIS. The two performance measurements adopted in this study are defined as follows:

(1) $$\mathrm{Sensitivity}=\frac{\mathrm{True}\;\mathrm{positive}}{\mathrm{True}\;\mathrm{positive}+\mathrm{False}\;\mathrm{negative}}$$

(2) $$\mathrm{Specificity}=\frac{\mathrm{True}\;\mathrm{negative}}{\mathrm{True}\;\mathrm{negative}+\mathrm{False}\;\mathrm{positive}}\;$$

The receiver operating characteristic (ROC) curves were also plotted and the area under the curves (AUC) was calculated as additional measurements of performance. To minimize possible false positives in the results, for each prediction, a threshold was adopted to guarantee that the specificity was no less than 99%. For each kinase, the optimal window size used to encode CMS encoding was determined using the best sensitivity obtained in LOOCV.

## Competing interests

The authors declare that they have no competing interests.

## Authors’ contributions

MHW and LZ conceived the idea of this work. MHW supervised the whole project and participated in its design and coordination. LZ and MHW developed the algorithm, implemented programs and drafted the major of the manuscript. MW developed the PKIS web server and helped to draft the manuscript. YS, JL and AL carried out the interpretation, reviewed the study and provided biological insights. All authors read and approved the final manuscript.

## Supplementary Material

Additional file 1: Table S1 Multivariate analysis of variance (MANOVA) of the CMS encoded features.In total, 50 kinases pass the statistical test as dictated by the requirements of MANOVA. Click here for file

Additional file 2: Figure S1 Comparison of PKIS with kinase-specific P-site prediction tools using the Phospho.ELM database. Some P-site prediction tools (such as KinasePhos) do not report scores for P-sites that are predicted to be unphosphorylated. To plot ROC curves, the scores of these P-sites were set at 0, which may sometimes lead to vertical ROC curves (dashed lines). Note that, in this case they may not precisely represent real performance of protein kinase identification processes. Click here for file

Additional file 3: Table S2 Cross-classification of specificity among 40 Ser/Thr kinases based on kinase identification models. Click here for file

Additional file 4: Table S3 Cross-classification of specificity among 16 Tyr kinases based on kinase identification models. Click here for file

Additional file 5: Figure S2 Sequence logos of amino acids surrounding phosphorylation sites catalysed by four kinases. The horizontal axis represents sequential positions relative to phosphorylation sites and the vertical axis represents decreases in uncertainty. Each letter denotes one amino acid. Click here for file

Additional file 6: Table S4 Cross-classification of performance of protein kinases with similar substrate motifs. Some kinase-specific P-site prediction tools (e.g. Musite) cannot distinguish different protein kinases within the same kinase group. For this reason, in this case, cross-classification is not applicable (NA). Click here for file

Additional file 7: Figure S3 A screen capture of a prediction made using PKIS. Two protein sequences were used in this example. PKIS also provides the CMS logo of each kinase, which contributes to better understanding of the substrate binding preference of each protein kinase. Click here for file

Additional file 8: Table S5 Statistics of all kinases in PKIS. The PKIS provides 56 kinases with more than 10 positive instances. Click here for file

Additional file 9: Figure S4 Difference between CMS and MS encoding strategies. Two different sequence encoding strategies were used. For the sake of simplicity and clarity, a sequence of 5 amino acids served as an example. Panel (A) shows the monomer spectrum (MS) encoding strategy. Panel (B) shows the composition of monomer spectrum (CMS) encoding strategy. Click here for file
