# Supplementary figures and images for: PKIS: computational identification of protein kinases for experimentally discovered protein phosphorylation sites
Source: BMC Bioinformatics. 2013 Aug 13;14:247. doi: 10.1186/1471-2105-14-247 (PMC3765618; doi:10.1186/1471-2105-14-247)

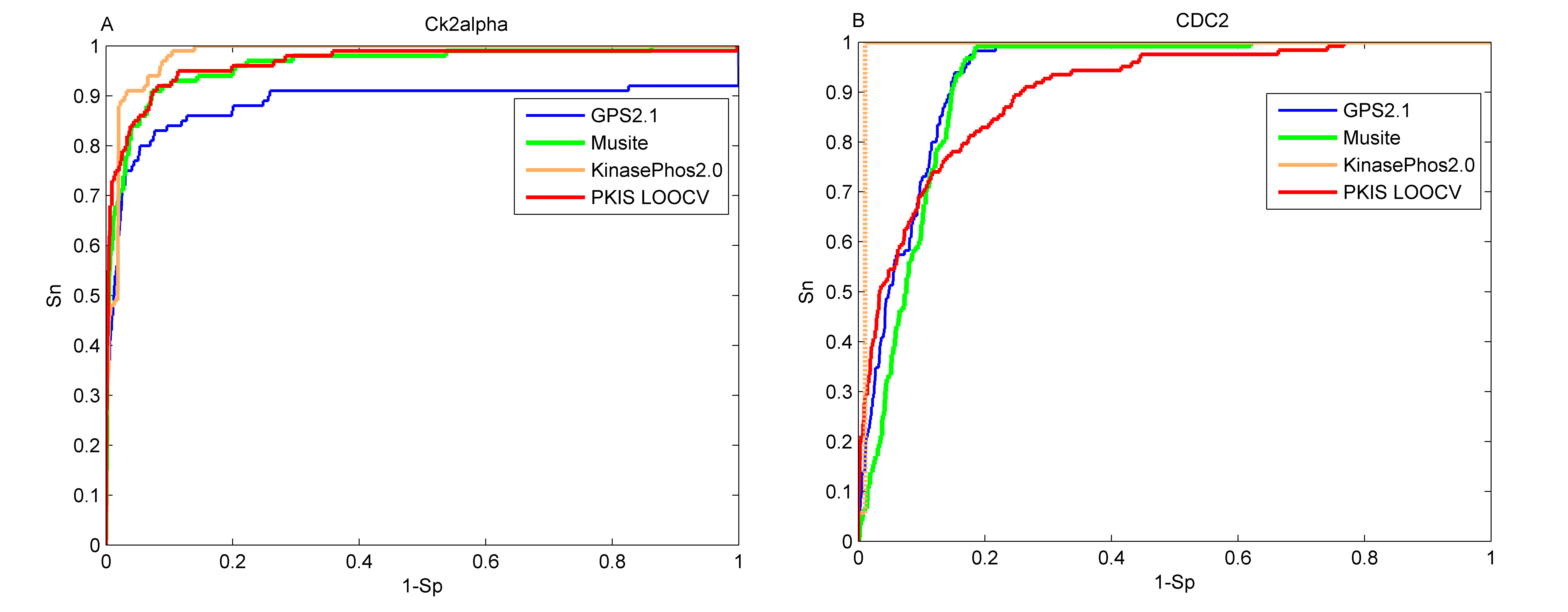

Supplement: Additional file 2: Figure S1 — Comparison of PKIS with kinase-specific P-site prediction tools using the Phospho.ELM database. Some P-site prediction tools (such as KinasePhos) do not report scores for P-sites that are predicted to be unphosphorylated. To plot ROC curves, the scores of these P-sites were set at 0, which may sometimes lead to vertical ROC curves (dashed lines). Note that, in this case they may not precisely represent real performance of protein kinase identification processes. [file 1471-2105-14-247-S2.jpeg]

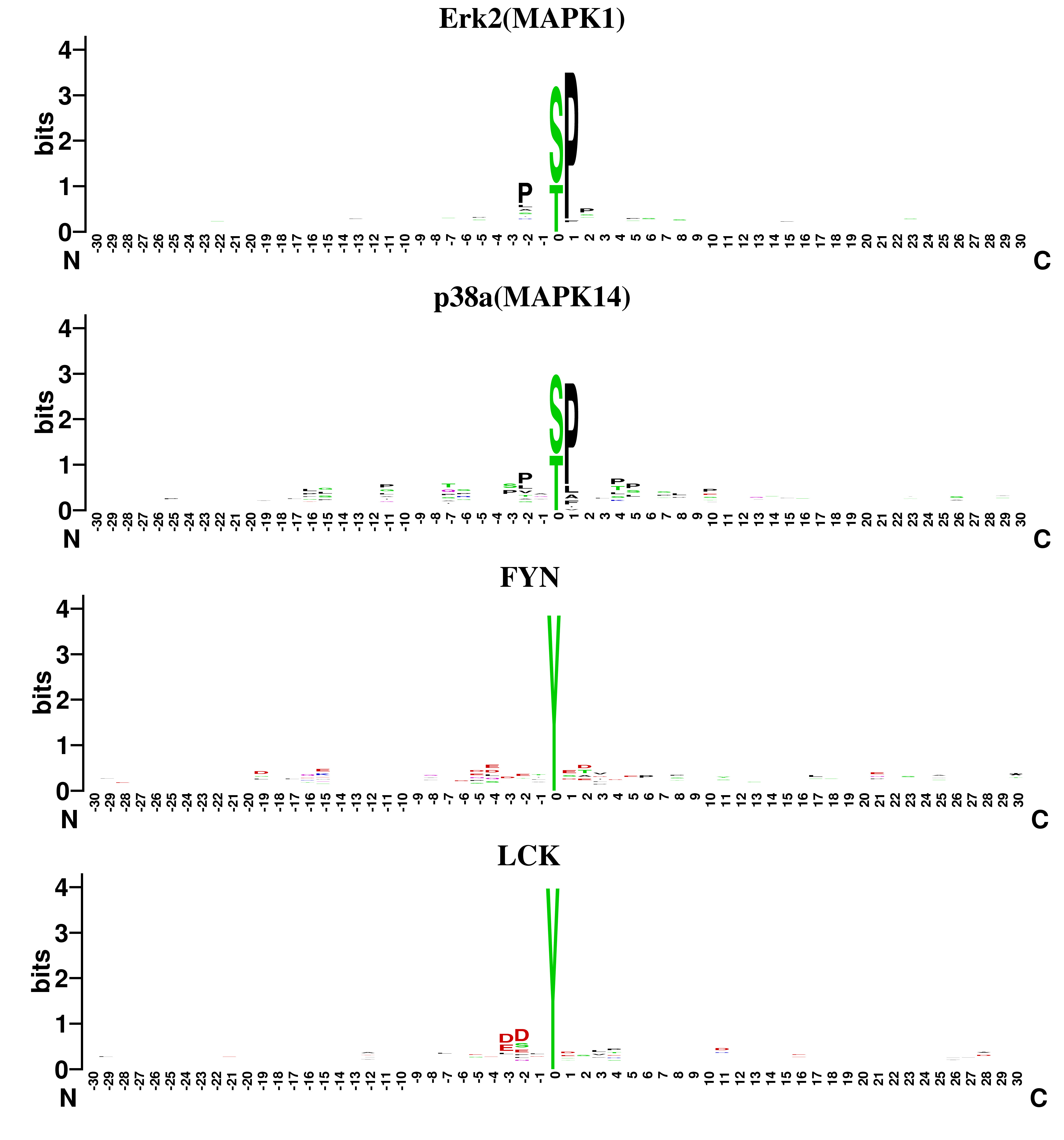

Supplement: Additional file 5: Figure S2 — Sequence logos of amino acids surrounding phosphorylation sites catalysed by four kinases. The horizontal axis represents sequential positions relative to phosphorylation sites and the vertical axis represents decreases in uncertainty. Each letter denotes one amino acid. [file 1471-2105-14-247-S5.jpeg]

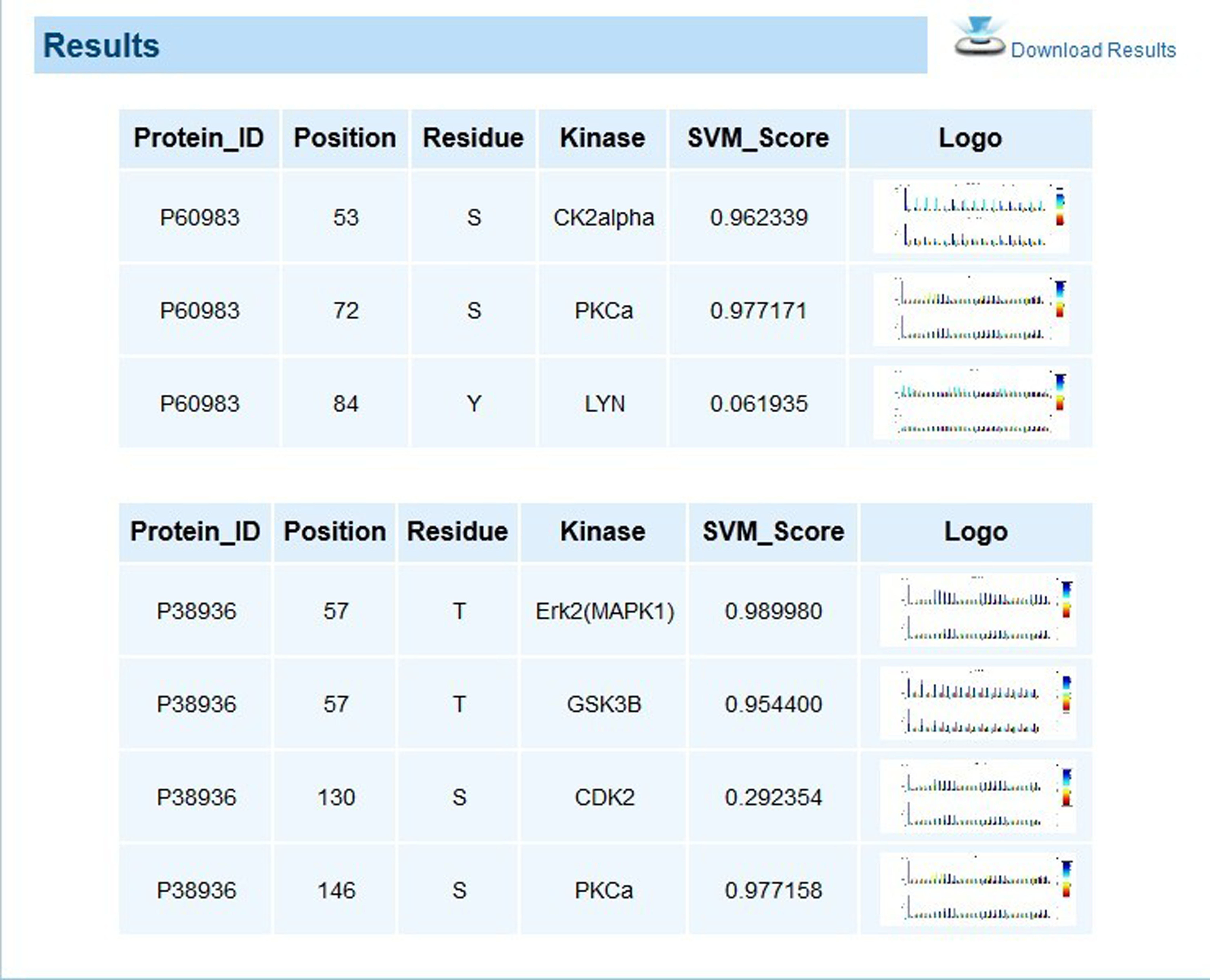

Supplement: Additional file 7: Figure S3 — A screen capture of a prediction made using PKIS. Two protein sequences were used in this example. PKIS also provides the CMS logo of each kinase, which contributes to better understanding of the substrate binding preference of each protein kinase. [file 1471-2105-14-247-S7.jpeg]

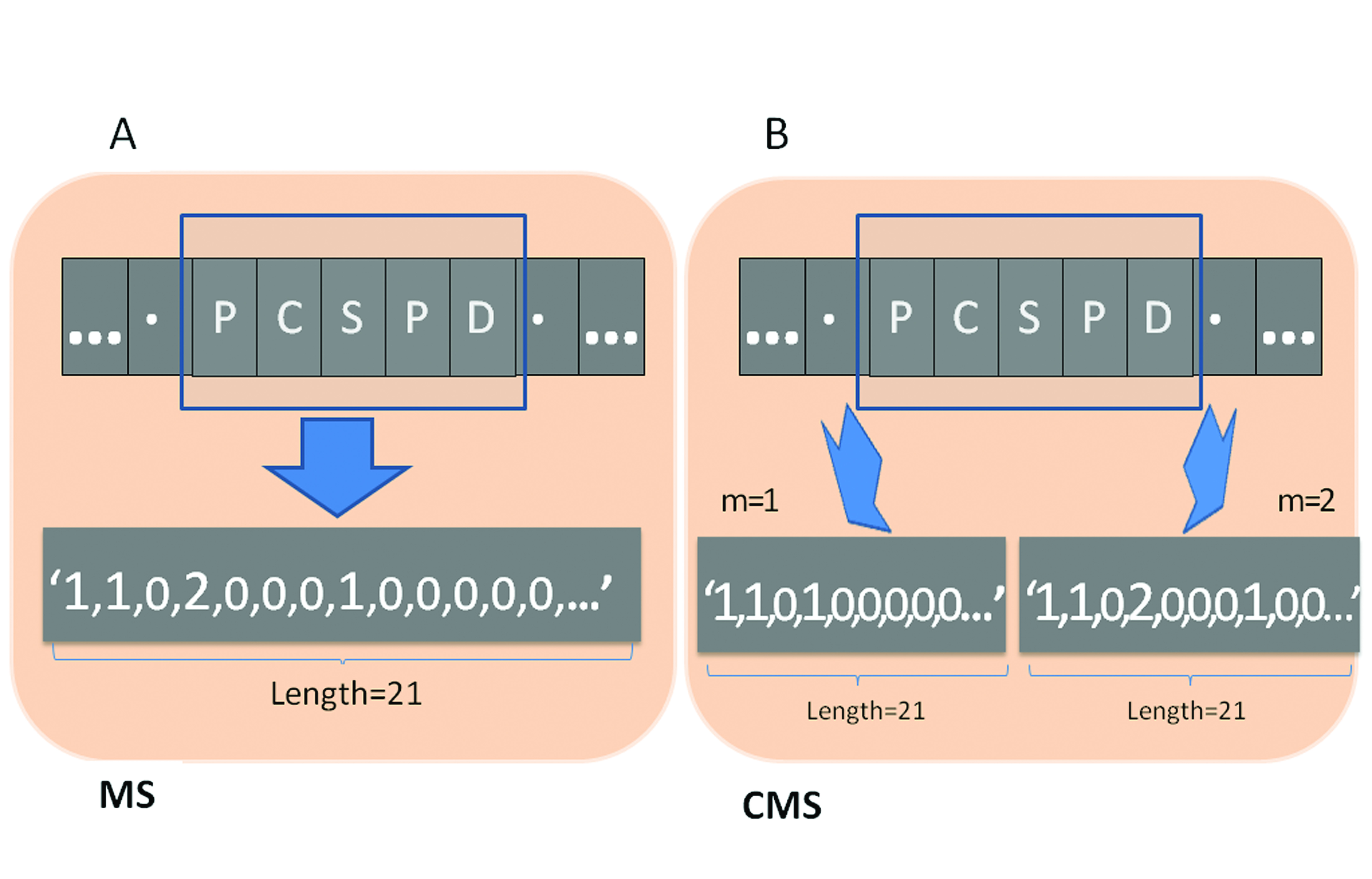

Supplement: Additional file 9: Figure S4 — Difference between CMS and MS encoding strategies. Two different sequence encoding strategies were used. For the sake of simplicity and clarity, a sequence of 5 amino acids served as an example. Panel (A) shows the monomer spectrum (MS) encoding strategy. Panel (B) shows the composition of monomer spectrum (CMS) encoding strategy. [file 1471-2105-14-247-S9.jpeg]
